# Prevalence and Social Acceptability of Cannabis, Tobacco, and Alcohol Use in Adult Women

**DOI:** 10.1089/whr.2021.0042

**Published:** 2021-10-04

**Authors:** Patricia Coughenour, Jaclyn S. Sadicario, Nicole Karjane, Anna Beth Parlier-Ahmad, Lisa Phipps, Dace S. Svikis

**Affiliations:** ^1^ Department of Obstetrics and Gynecology, Virginia Commonwealth University, VCU Health, Richmond, Virginia, USA.; ^2^ Department of Psychology, Virginia Commonwealth University, Richmond, Virginia, USA.; ^3^ Institute for Women's Health, Virginia Commonwealth University, VCU Health, Richmond, Virginia, USA.

**Keywords:** women, social acceptability, attitudes, cannabis, tobacco

## Abstract

***Background:*** As more US states legalize cannabis use, prevalence of use continues to rise and attitudes toward use are changing. This study examined (1) the relationship between cannabis use and social acceptability of use and (2) how social acceptability and use of cannabis relate to anxiety, depression, and several pain conditions.

***Materials and Methods:*** Participants were *n* = 210 nonpregnant women recruited from two women's health clinics for an anonymous survey of complementary and integrative health practices. Survey domains included demographics, recent and lifetime cannabis, cigarette, and alcohol use, depression, anxiety, pain, and social acceptability of substances studied.

***Results:*** The sample had a mean age of 38.7 years and was 50.0% Black. Approximately 12.9% of the sample endorsed recent cannabis use, 17.2% endorsed recent cigarette use, and 57.5% endorsed recent alcohol use. Acceptability of use varied by substance. One-third (33.3%) of women found cannabis use to be socially acceptable. Higher social acceptability scores for cannabis were correlated with higher acceptability scores for each of the other substances studied, with the strongest correlation for e-cigarettes (*R*^2^ of 0.395, *p* < 0.001) and the weakest for alcohol (*R*^2^ of 0.296, *p* < 0.001). Women reporting anxiety (38.9%) and recent acute pain (28.6%) rated cannabis use as more socially acceptable than those without such symptoms.

***Conclusions:*** Women with recent cannabis use were more likely to find use of alcohol, tobacco, and cannabis to be socially acceptable than those not reporting cannabis use. More research is needed to better understand these relationships, as they might help to identify opportunities for education and intervention in this population.

## Introduction

Trends in cannabis use have varied over time. Since the 1990s, rates of use have increased by more than 20%.^1^ From 2006 to 2014, the number of daily users 12 years of age and older has nearly doubled. One potential contributor to the increased prevalence of cannabis use may be the lack of perceived harm associated with use.^2–4^ In addition, recent changes in laws governing cannabis use and sale, including legalization in many states, and relative decriminalization in several others, may have contributed to an increase in social acceptability of cannabis use.^5^

Concurrent with such changes in policy and attitudes, the United States has experienced a rise in rates of cannabis use. From 2008 to 2016, these patterns were more pronounced in states that had legalized medical cannabis before 2006.^6^ Thus, it is important to better understand the relationship between social acceptability and use of cannabis.

To date, much research investigating use of, attitudes toward, and health effects of cannabis have focused on adolescents and findings show that rates of use among those 12–17 years of age remain steady.^5^ However, among samples older than 18 years, cannabis use has increased. Studies of cannabis use in the adult population have focused predominately on males.^7^ Although this is likely due, in part, to data suggesting that rates of use are higher among men than women,^8^ there has also been a historical bias toward conducting research with male participants over female ones.^9^ As a result, there has been little research into attitudes about cannabis use and how they relate to patterns of cannabis use in adult women.

Understanding attitudes toward cannabis use is important given the potential adverse consequences of use, including impairment of short-term memory and motor coordination, altered brain development, and cardiovascular and pulmonary complications.^10^ Understanding prevalence of cannabis use in adult women and their attitudes toward use is particularly important, as some studies suggest potential differential health effects of cannabis in women.^11^ Research also suggests that individuals who use cannabis may be doing so to “treat” symptoms of anxiety and depression, and/or pain severity (acute, chronic, and migraines).^12,13^

This study describes current rates of cannabis use and social acceptability ratings for cannabis and other drugs in a sample of adult women. It also examines how social acceptability and use of cannabis relate to conditions commonly associated with cannabis use, including anxiety, depression, and pain.^12^ Data analyses tested two hypotheses: (1) ratings of social acceptability for cannabis use will be higher in women with recent cannabis use compared to women without such use and (2) women experiencing depression, anxiety, or pain will be more likely to report recent cannabis use than women not experiencing these conditions.

## Materials and Methods

Participants were nonpregnant women (*N* = 210) receiving care at one of two women's health clinics in central Virginia. We invited participants to complete a 15-minute, electronic, anonymous survey about complimentary and integrative health practices (CIHP).^14^ Items focused on use of CIHP like acupuncture, supplements, and hypnosis, as well as alcohol, cannabis, and other drug use. Participants were able to skip any question they chose not to answer.

This study focused on recent and lifetime substance use and attitudes about use as well as anxiety, depression, and pain. Recruitment took place between September 2017 and July 2019. Participation was voluntary, and participants received a $5 gift card for their participation. The research was reviewed and approved by the VCU Institutional Review Board, and all women provided informed consent for study participation.

### Measures

Demographic variables included age, race, ethnicity, marital status, education, and current employment status.

Participants answered a series of yes/no questions related to the use of the following substances: cigarettes, e-cigarettes, other tobacco products (cigars, chewing tobacco, dip, hookah, and pipe tobacco), alcohol, and cannabis/marijuana. Both lifetime and recent use were assessed with separate questions. For example: “Have you ever used [substance]?” and “Have you used [substance] recently (within the last 30 days)?”

Social acceptability of use of substances was measured with one item adapted from Berg et al. (2015)^15^ asking “How socially acceptable do you find [substance] use?” This item was asked separately for cigarettes, e-cigarettes, other tobacco, alcohol, and cannabis. Response options ranged from 1 = totally unacceptable to 7 = perfectly acceptable, with 4 = neutral.

Anxiety and depression were each assessed with a self-report as well as a standardized screening tool. The self-report question was a single yes/no item (“Do you have anxiety?” and “Do you have depression?”).

The General Anxiety Disorder-7 (GAD-7), a standardized tool with good psychometric properties,^16^ was used to assess current (within the past 2 weeks) symptoms of anxiety. According to the guidelines for using the GAD-7, scores from 0 to 9 were considered a negative screen, while scores ≥10 were considered a positive screen, indicative of current, clinical symptoms of anxiety.

The Patient Health Questionnaire-9 (PHQ-9), a standardized tool developed by Kroenke et al.,^17^ was used to assess current (within the past 2 weeks) symptoms of depression. According to the guidelines for using the PHQ-9, scores from 0 to 9 were considered a negative screen, while scores ≥10 were considered a positive screen, indicative of current, clinical symptoms of depression.

A series of yes/no questions was asked about recent (past 30 days) and lifetime (any) migraine, as well as acute and chronic pain.

### Data analysis

Descriptive statistics were used to characterize the sample. Data were analyzed using Pearson chi-square for categorical variables and *t*-tests or Mann-Whitney U for continuous measures, as appropriate for normality. Because social acceptability scores were not normally distributed, the nonparametric Mann-Whitney U test was performed to examine differences in social acceptability of cannabis across medical conditions. Fisher's exact test was used when expected cell counts were <5.

The Spearman's correlation coefficient was used to examine the relationship between social acceptability of cannabis use and that of other substances. A significance threshold of 0.05 was used throughout this study. Participants who did not answer lifetime use questions (for any substance) or lifetime experience questions (for anxiety, depression, or pain) were excluded pairwise for analysis. Because both GAD-7 and PHQ-9 are cumulative scores, participants who did not answer all questions within each scale were excluded from those analyses. Adjusted sample sizes for these analyses are noted in each table.

## Results

### Demographics

The sample of *N* = 210 nonpregnant women had a mean age of 38.7 years (SD = 11.9). As shown in Table 1, the sample was primarily black (50.0%) or white (46.2%), and most women were working full time (59.0%), had at least an undergraduate degree (52.9%), and were not currently married (66.7%). Over one-fourth of women reported having depression (27.5%), and over one-third reported having anxiety (38.9%).

**Table 1.** Participant characteristics, health, and substance use history

Variable	Total *N* = 210
Age (years), *M* (SD)	38.7 (11.9)
Race, *N* (%)	
White	97 (46.2)
Black	105 (50.0)
Other	8 (3.80)
Bachelor's degree, *N* (%)	
<BA	99 (47.1)
≥BA	111 (52.9)
Marital status, *N* (%)	
Currently married	70 (33.3)
Single/never married	98 (46.7)
Employment, *N* (%)	
Full time	124 (59.0)
Part time	27 (12.9)
Unemployed	39 (18.6)
Self-report, *N* (%)	
Anxiety^a^	81 (38.9)
Depression^b^	57 (27.5)
Migraines^c^	71 (34.0)
Recent chronic pain^d^	67 (76.1)
Recent acute pain^d^	60 (56.1)
Current symptoms, *N* (%)	
Anxiety^e,f^	26 (13.7)
Depression^g,h^	22 (12.0)
Substance use, *N* (%)	
Cannabis: lifetime	77 (36.7)
Recent	27 (12.9)
Alcohol^b^	179 (86.5)
	119 (57.5)
Cigarette^c^	83 (39.7)
	36 (17.2)
e-Cigarette^i^	35 (17.0)
	8 (3.9)
Other tobacco^c^	23 (11.0)
	3 (1.4)
Social acceptability, median (25th, 75th percentiles)	
Cannabis	4 (2, 6)
Alcohol	6 (4, 6)
Cigarettes	2 (1, 4)
e-Cigarettes	3 (1, 4)
Other tobacco	3 (1, 4)

^a^ *N* for this analysis = 208.

^b^ *N* for this analysis = 207.

^c^ *N* for this analysis = 209.

^d^ Recent defined as use within the past 30 days.

^e^ Positive screen for current anxiety symptoms on GAD-7.

^f^ *N* for this analysis = 190.

^g^ Positive screen for current depression symptoms on PHQ-9.

^h^ *N* for this analysis = 183.

^i^ *N* for this analysis = 206.

BA, bachelor's degree; GAD-7, general anxiety disorder-7; PHQ-9, patient health questionnaire-9.

Approximately 13.7% of participants screened positive for current symptoms of anxiety on the GAD-7 and 12.0% screened positive for current symptoms of depression on the PHQ-9. Over one-third (34.0%) of women reported having migraines (lifetime), and over half reported experiencing recent acute (28.6%) and/or chronic (31.9%) pain.

Alcohol was the most commonly used substance with 85.5% of women reporting any use in their lifetime. For both cigarettes (tobacco) and cannabis, close to one-third of women endorsed lifetime use (39.7% and 36.7%, respectively). Similarly, alcohol was the most common recently used substance (57.5%), followed by 17.2% and 12.9% of women reporting recent use of cigarettes and cannabis, respectively. Rates of recent e-cigarette (3.9%) and other tobacco product (1.4%) use were even lower (Table 1).

Alcohol was the substance with the highest median social acceptability score followed by cannabis and finally cigarettes (Table 1).

### Correlates of cannabis use

Women with and without recent cannabis use did not differ in self-reported anxiety or depression, current anxiety, or depressive symptoms based on GAD-7 and PHQ-9 scores, or reports of recent or lifetime acute or chronic pain (all *p* > 0.05) (Table 2).

**Table 2.** Anxiety, depression, and pain symptoms in women with and without recent cannabis use

Medical condition	Recent cannabis use^a^ *N* (%) *N* = 27	No recent cannabis use^a^ *N* (%) *N* = 182	*p*
Self-report			
Anxiety^b^	11 (40.7)	70 (38.7)	0.837
Depression^c^	10 (37.0)	47 (26.1)	0.236
Migraines^d^	5 (18.5)	66 (36.3)	0.069
Recent chronic pain^a^	8 (29.6)	59 (32.4)	0.772
Recent acute pain^a^	9 (34.6)	51 (28.2)	0.499
Current symptoms			
Anxiety^e,f^	6 (23.1)	20 (12.2)	0.134
Depression^g,h^	5 (19.2)	17 (10.8)	0.209^i^

^a^ Recent defined as presents within past 30 days.

^b^ *N* for this analysis = 208

^c^ *N* for this analysis = 207.

^d^ *N* for this analysis = 209

^e^ Positive screen for anxiety symptoms on GAD-7.

^f^ *N* for this analysis = 190.

^g^ Positive screen for depression symptoms on PHQ-9.

^h^ *N* for this analysis = 183.

^i^ *p*-value from Fisher's exact test due to low expected cell count.

### Social acceptability and cannabis use

The median score for the social acceptability of cannabis use was 4.0 out of a maximum of 7 (SD 2.0). Approximately one-third of participants rated cannabis use as acceptable (*N* = 70), one-third were neutral (*N* = 65), and the remaining one-third found use unacceptable (*N* = 72). Women with recent cannabis use found cannabis use more acceptable [*M* = 5.9 (SD 1.4)] compared to those with no recent use [*M* = 3.6 (SD 1.9)] (*t* = 7.451; *p* < 0.001). Woman with a history of anxiety had more favorable attitudes toward cannabis use than those without (*p* = 0.008) (Table 3). In addition, women reporting recent acute pain found cannabis use more socially acceptable than those who did not (*p* = 0.005). No other between group difference was found.

**Table 3.** Social acceptability of cannabis use by commonly associated conditions

Medical condition	Yes (mean rank)	No (mean rank)	Standardized test statistic	*p*
Self-report				
Anxiety^a^	116.8	94.9	2.637	**0.008**
Depression^b^	115.5	98.2	1.922	0.055
Migraines^c^	106.9	102.4	0.529	0.597
Recent chronic pain^d^	107.7	102.3	0.617	0.537
Recent acute pain^d^	120.5	95.7	2.784	**0.005**
Current symptoms				
Anxiety, GAD-7^e,f^	102.7	93.2	0.846	0.398
Depression, PHQ-9^g,h^	103.6	89.3	1.231	0.218

Bold values indicate the significant at *p* < .008.

^a^ *N* for this analysis = 208.

^b^ *N* for this analysis = 207.

^c^ *N* for this analysis = 209.

^d^ Recent defined as use within past 30 days.

^e^ Positive screen for anxiety symptoms on GAD-7.

^f^ *N* for this analysis = 190.

^g^ Positive screen for depression symptoms on PHQ-9.

^h^ *N* for this analysis = 183.

### Correlation between social acceptability of cannabis use and other substance use

As shown in Table 4, higher social acceptability scores for cannabis were correlated with higher acceptability scores for each of the other substances studied, with the strongest correlation for the use of e-cigarettes (*R*^2^ of 0.395, *p* < 0.001) and the weakest for alcohol (*R*^2^ of 0.296, *p* < 0.001).

**Table 4.** Social acceptability of other substance use by social acceptability of cannabis use

Substance	R^2^	*p*
Cannabis	—	—
Alcohol	0.296	<0.001
Cigarette	0.344	<0.001
e-Cigarette	0.395	<0.001
Other tobacco	0.319	<0.001

## Discussion

This study examined the prevalence of cannabis use and social acceptability of such use in adult women—an understudied group, despite increasing rates of cannabis use. As hypothesized, social acceptability ratings for cannabis were more favorable among women with recent use compared to those without such use. In contrast, hypothesis two was not supported by the data, as there was no relationship between cannabis use and anxiety, depression, and/or pain. However, this study did find relationships between both anxiety and acute pain and social acceptability rates for cannabis use. Specifically, social acceptability ratings for cannabis use were higher for women who reported anxiety or acute pain than for women who did not endorse those conditions.

Women found cannabis use to be more socially acceptable than cigarette, e-cigarette, and other tobacco use, although less acceptable than alcohol use. In addition, participants who found cannabis use more acceptable also found cigarette, e-cigarette, and alcohol use more acceptable than the women who rated cannabis use as unacceptable. Previous research has shown that such attitudes may translate into engaging in the use of those substances.^18^ The results may suggest that social acceptability of certain substances may be used to predict the use of other drugs and risky behaviors.

The findings are congruent with recent data that suggest attitudes toward cannabis use are becoming more favorable with changing laws and policy.^19^ In contrast, tobacco use was seen as socially unacceptable, which is likely due, in part, to the decades of advertising and policy changes that increased public awareness of the negative health effects of tobacco use.^20^

Previous research has shown that social acceptability, along with perception of risk, correlates with behavior choices. Those who find a behavior more socially acceptable are more likely to engage in that behavior.^8^ The findings of this study suggest that there are significant rates of cannabis use among adult women and that women who report a history of anxiety may be more likely to see cannabis use as acceptable and therefore more likely to engage in use of the substance.

However, results suggest the relationship is complex. While a yes/no question about history of anxiety was associated with higher acceptability ratings for cannabis use, a standardized screen for current, clinical anxiety (defined as GAD-7 scores >10) was unrelated to these attitudes. This suggests that these results are likely impacted by how anxiety is assessed; the single-item anxiety question did not specify a minimum severity level or a time period (recent or lifetime), while the GAD-7 is a multiquestion, well-studied screen for current symptoms.

Women who reported recent acute pain (past 30 days) also found cannabis use more acceptable than their counterparts who did not endorse such pain. This trend, however, was limited to acute pain and was not found for women experiencing ongoing chronic pain. While these findings are consistent with previous research, they should be interpreted with caution due to the small portion of the sample endorsing pain. Prior research on attitudes toward cannabis use have found pain to be correlated with more positive views of cannabis use,^21^ but more research is needed to further parse out attitudes in those with chronic versus acute pain.

Changing legal and social policies on medical and recreational cannabis use affirm the need for further research to better understand perceptions of the use of cannabis in this population. The finding that women rated cannabis use more socially acceptable than e-cigarette and cigarette use may indicate less perceived harm from cannabis use than cigarette use among study participants. This may point to a need for education on risks of use across substances.

Risks of cannabis use are not isolated to the method of use. Smoking cannabis can have similar negative health effects to cigarettes. Edible forms of cannabis may not incur the same risk as smoking; however, it may have a higher concentration of cannabinoids, potentially increasing risk for other health issues.^22^ Independent of those risks, several studies have also shown that cannabis may contribute to or exacerbate mental health symptoms. In addition, cannabis use in women of childbearing age may present additional risks and considerations.

Although specific relationships between cannabis use and health effects in the perinatal period are not clear in the literature, several studies have shown there may be an increased risk related to cannabis use during pregnancy or while breastfeeding,^23,24^ with many professional organizations advising against use during the perinatal and postpartum periods (ACOG, American Academy of Pediatrics). Furthermore, health systems have erred on the side of caution and prohibited breastfeeding on the mother baby units in mothers who have endorsed cannabis use. Given the known benefits of breastfeeding over formula feeding, further research is needed to understand relative risks of harm with cannabis use. It is imperative that both clinicians and researchers gain a clear understanding of cannabis use patterns in adult women so that health care providers can better identify and serve patients at increased risk with effective screening and education practices.

More research is needed to replicate and understand this study's findings. Strengths of this study include the use of anonymously collected survey data, which are often associated with higher rates of disclosure because of reduced fear of social stigma and negative consequences.^25^ In addition, the majority of participants were black women. Hence, we hope this study's findings can contribute to a growing body of knowledge in health disparities research within historically understudied groups whose experiences of discrimination may lead to them being reluctant to participate in clinical research.^26^

Several limitations should be also noted. First, all data were self-report, and women may have underreported their use of cannabis and other substances. During the period of data collection for this study in Virginia, medical cannabis use was legal, but limited to a very small subgroup of seriously ill individuals and to specific forms of cannabis. More recently (July 1, 2021), recreational cannabis use was legalized, affirming the need for ongoing research on changing attitudes and perceptions.^27^ Second, participants in this study were recruited from clinics in central Virginia and were seeking medical care. Thus, the sample is likely not generalizable to the general population of the United States and more rural settings.

Third, questions about the use of cannabis and other substances were asked as part of a survey primarily focused on the use of complementary and integrative therapies, which may have changed reported use and/or social acceptability rankings, as use in the context of a medical condition may be seen more favorably. The parent survey was not specific to substance use or cannabis use. Fourth, the questions about recent substance use did not ask about quantity or frequency of use and could reflect anything from a single occasion to daily use. Future research should specifically examine prevalence and attitudes in women with cannabis or other substance use disorders in depth, with social use and people who use cannabis chronically examined separately.^28^

It should also be noted that questions on cannabis use did not differentiate between use of the marijuana plant versus cannabidiol (CBD)-containing products, which have become more prevalent over the last several years and do not appear to cause dependence or other health-related problems. Use of different cannabinoids, including CBD products, may be the subject of future research efforts.

## Conclusions

This study examined use rates and social acceptability of cannabis in women in Central Virginia. It also examined associations between cannabis use and social acceptability of cannabis use, as well as differences in acceptability among women with commonly associated conditions such as anxiety, depression, and pain. This study also compared ratings of acceptability for cannabis use to ratings of acceptability for various tobacco products and alcohol. Although alcohol was found to be the most socially acceptable substance, cannabis was perceived to be more acceptable than all forms of tobacco studied, including cigarettes and e-cigarettes. In this study, no relationship was found between cannabis use and reports of anxiety, depression, and pain, conditions sometimes associated with use. However, women who reported a history of anxiety rated cannabis use as more acceptable than those without a history of anxiety. Women who found cannabis use to be acceptable also found the use of alcohol and tobacco products to be more acceptable. Future research should characterize these trends and investigate similar trends and attitudes toward cannabis use among women, including the perinatal and postpartum period, because this is a developing area with the potential to impact health care policy and law.

## Abbreviations Used

CBD: cannabidiol
CIHP: complimentary and integrative health practices
GAD-7: general anxiety disorder-7
PHQ-9: patient health questionnaire-9
